# Durum Wheat Products—Recent Advances

**DOI:** 10.3390/foods11223660

**Published:** 2022-11-16

**Authors:** Mike Sissons

**Affiliations:** NSW Department of Primary Industries, Tamworth Agricultural Institute, 4 Marsden Park Road, Calala, NSW 2340, Australia; mike.sissons@dpi.nsw.gov.au

Durum wheat is widely used in various products, including long and short dried pasta, fresh and sheeted pasta, couscous, bulgur and baked bread. The quality of the raw material, durum wheat, is critical for ensuring that these products meet their specifications. While the genotype chosen and environmental conditions during crop development are important for determining the final grain quality, its processing by milling, sizing or mixing and the end-product manufacture are important processes affecting the end product’s quality. The key features of grains that strongly influence the yield are a good kernel size, high test weight, and high percentage of hard vitreous kernels, with minimal contaminants and no surface discoloration. While large grains provide more semolina, kernel-size uniformity is very important in the milling process. Wang et al. [1] evaluated the influence of kernel size and its potential interaction with genotype on key quality traits of durum wheat. Genotype was shown to have a strong impact on the test weight of small kernels and the milling yields of medium and large kernels. Millers are now striving to increase productivity, ensure food safety, and enhance sustainability efforts; for example, three studies on the better use of lower-value milling wastes (bran and germ) demonstrate the use of durum oil in focaccia to improve its resistance to oxidation [2], its use in biscuit making to decrease oxidative phenomena and increase bioactives [3], and its use to improve the stabilization of germ, to exploit the nutrients and bioactive compounds within wheat germ [4]. The key indicators of milling quality are the yields (total and semolina), ash content, and speck counts in the finished granular product. Recent milling developments in processing equipment and digital applications to improve quality and sustainability are reviewed by Sarkar and Fu [5]. Other durum wheat products are also considered, such as couscous, a product prepared from durum wheat semolina that agglomerates upon the addition of water and undergoes physical and thermal treatment. Its history, its manufacture on traditional and industrial scales, and its consumption in traditional and modern ways are reviewed by Hammami et al. to supplement the lack of scientific and technical descriptions for couscous [6]. Pasta makers require high-quality semolina meeting their industrial requirements, so methods to ascertain semolina quality are critical. The main factors determining the technological quality of semolina and approaches used for evaluating gluten quality are reviewed by Cecchini et al. [7]. There is a lack of standard tests across the industry; the tests have historically been adapted from methods for evaluating bread wheat and largely based on laboratory rather than industrial evaluation, so a better test to determine the behaviour of durum wheat semolina and the cooking quality of its corresponding pasta needs to be developed. Producing pasta while mostly using a few ingredients such as semolina, water and, perhaps, eggs is a complex process with many process variables. A change in a single variable, such as the type of raw material (refined vs. wholegrain semolina), can affect the entire process and product quality. Understanding the relationship between the processing variables and pasta quality is essential in “redesigning” the process when alternative raw materials (i.e., ingredients other than durum wheat semolina) are used [8]. An example is optimising the drying process for the manufacture of bulgur from grains of different quality to optimise the phytonutrient content [9].

There is a new trend in food consumption, especially in well-developed economies. Many consumers are increasingly interested in food that provides a benefit in preventing or reducing nutrition-related diseases, so-called “lifestyle or civilisation” diseases such as cancer, diabetes, cardiovascular disease, and obesity. These diseases afflict a large percentage of the population of Westernised countries, with the trend continuing to worsen in developing nations, and are the main non-communicable causes of death. Manufacturers are responding by trying to improve nutritional value or create presumed health benefits. Over the past decade or two, many studies have largely focused on a partial substitution of semolina or flour to create pasta products with improved nutritional value, such as higher fibre, better protein quality, and enrichment in essential fatty acids. However, few of these novel pasta products have shown clinical benefits. Sissons provides an update on durum-derived pasta products with proposed health benefits [10], while Di Pede et al. [11] provide an overview of the glycaemic indices of different pasta formulations. A new focus on algae as a food ingredient has been growing in Western diets. Macroalgae or seaweeds are low-calorie ingredients that are high in protein and iodine, low in fat, and a source of hydrocolloids, minerals, vitamins, and bioactive compounds (antioxidants). *Fucus vesiculosus*, a brown macroalga, was added to a pasta recipe, but, as is often the case, even relatively low incorporation impaired the quality of the pasta [12]. The issue with limited incorporation, which can limit protein and fibre enrichment, was investigated by Martín-Esparza et al. [13] in fresh tiger-nut-based tagliatelle using hydrocolloids as a structural agent to improve the textural characteristics, cooking properties, and fibre loss. Another approach to issues regarding substitution limits is described in a report on pasta enriched with encapsulated carrot waste; this waste is rich in carotenoids, and the encapsulation protects the pigments from thermal degradation. This resulted in pasta with significantly improved protein, fat, and ash contents [14]. An alternative approach to substituting semolina to create novel health-promoting pasta is the genetic manipulation of the starch and protein composition and modification of the hardness of durum wheat in order to improve its nutritional and technological value and expand its utilization for application to a wider range of end products. Increasing the amylose content, moving glutenin genes from bread wheat into durum to improve its bread-making performance, and making the kernel softer for use in biscuits, pizza, and other foods are the approaches reviewed by Lafiandra et al. [15].
